# Genome-wide association study identifies a maternal copy-number deletion in *PSG11* enriched among preeclampsia patients

**DOI:** 10.1186/1471-2393-12-61

**Published:** 2012-06-29

**Authors:** Linlu Zhao, Elizabeth W Triche, Kyle M Walsh, Michael B Bracken, Audrey F Saftlas, Josephine Hoh, Andrew T Dewan

**Affiliations:** 1 Center for Perinatal, Pediatric and Environmental Epidemiology, Yale School of Public Health, New Haven, CT, USA; 2 Department of Epidemiology, Brown University School of Medicine, Providence, RI, USA; 3 Department of Epidemiology, University of Iowa College of Public Health, Iowa City, IA, USA; 4 Division of Environmental Health Sciences, Yale School of Public Health, New Haven, CT, USA; 5 Division of Chronic Disease Epidemiology, Yale School of Public Health, 60 College Street, Room 403, New Haven, CT, 06520, USA

**Keywords:** Copy-number variant, Genome-wide association study, Microarray analysis, Preeclampsia, Single nucleotide polymorphism

## Abstract

**Background:**

Specific genetic contributions for preeclampsia (PE) are currently unknown. This genome-wide association study (GWAS) aims to identify maternal single nucleotide polymorphisms (SNPs) and copy-number variants (CNVs) involved in the etiology of PE.

**Methods:**

A genome-wide scan was performed on 177 PE cases (diagnosed according to National Heart, Lung and Blood Institute guidelines) and 116 normotensive controls. White female study subjects from Iowa were genotyped on Affymetrix SNP 6.0 microarrays. CNV calls made using a combination of four detection algorithms (Birdseye, Canary, PennCNV, and QuantiSNP) were merged using CNVision and screened with stringent prioritization criteria. Due to limited DNA quantities and the deleterious nature of copy-number deletions, it was decided *a priori* that only deletions would be selected for assay on the entire case-control dataset using quantitative real-time PCR.

**Results:**

The top four SNP candidates had an allelic or genotypic *p*-value between 10^-5^ and 10^-6^, however, none surpassed the Bonferroni-corrected significance threshold. Three recurrent rare deletions meeting prioritization criteria detected in multiple cases were selected for targeted genotyping. A locus of particular interest was found showing an enrichment of case deletions in 19q13.31 (5/169 cases and 1/114 controls), which encompasses the *PSG11* gene contiguous to a highly plastic genomic region. All algorithm calls for these regions were assay confirmed.

**Conclusions:**

CNVs may confer risk for PE and represent interesting regions that warrant further investigation. Top SNP candidates identified from the GWAS, although not genome-wide significant, may be useful to inform future studies in PE genetics.

## Background

Preeclampsia (PE) is a pregnancy-specific complication which affects 2-7% of pregnancies [1]. Recognized as a leading cause of maternal and fetal morbidity and mortality worldwide, PE is characterized by new onset hypertension and proteinuria with or without other multi-system disorders. Family-based studies in several geographically and ethnically diverse populations have demonstrated the familial nature of PE [2-5]. Despite the evidence for a genetic basis of PE, its exact etiology remains undefined.

Numerous candidate genes have been implicated in the pathogenesis of PE; particularly genes involved in immune maladaptation, placental ischemia, and increased oxidative stress [6]. Recent research found that the complement system may play a role in PE [7]. While sequence variants in over 70 genes have been investigated, the majority of studies have focused on only a handful of these genes [8,9]. Hundreds of candidate gene studies have been conducted, but findings have been inconsistent [10]. Selection of candidate genes for investigation is limited by an incomplete understanding of biological processes involved in the pathogenesis of PE [8]. Furthermore, most existing studies examining the genetic basis of PE have focused on single nucleotide polymorphisms (SNPs), which explain only a small proportion of the overall heritability of complex disorders.

Copy-number variants (CNVs), another type of genetic variation, may contribute to the missing heritability of complex disorders such as PE [11]. An estimated 13% of the human genome is believed to be copy-number variable [12]. These variants range from less than one kilobase (kb) to several megabases (Mb) in size and include deletions and amplifications [13]. Functionally relevant CNVs can alter gene function or regulation by various proposed mechanisms, including alteration of gene dosage, gene interruption, gene fusion, and alteration of a gene’s position relative to regulatory elements. CNVs may induce phenotypic changes. The phenotypic consequences of these alterations depend on the nature and extent of the deleted or duplicated DNA sequence [14,15]. The disruption of genes by CNVs has been linked to numerous complex disorders, including neuropsychiatric and autoimmune disorders [16]. However, to date there have been no reports examining an association between CNVs and PE.

Given the current limited state of knowledge on the genetics of PE, a genome-wide association study (GWAS) was conducted to identify potential PE-associated SNPs and CNVs using a case-control study design. This is the first reported GWAS on PE.

## Materials and methods

### Ethics statement

This study was approved by the University of Iowa Institutional Review Board and the Yale University Human Investigation Committee.

### Study population

Female subjects were recruited through the SOPHIA study—a case-control study designed to examine the roles of maternal-fetal human leukocyte antigen and sexual history in PE [17]. A total of 3078 primaparous mothers who gave birth in Iowa from August 2002 to May 2005 were identified from electronic birth certificates provided by the Iowa Department of Public Health as potential subjects. Potential PE cases were selected from primiparous women who were “check-box positive” on their infant’s birth certificate for pregnancy-induced hypertension or eclampsia. Potential controls were a random sample of primiparas who had no indication of hypertension on their infant’s birth certificate. Willing subjects were screened for initial eligibility and excluded based on any of the following criteria: age <18 years at delivery; non-English-speaking; history of an autoimmune disease (e.g. systemic lupus erythematosus, insulin-dependent diabetes mellitus, rheumatoid arthritis); recurrent spontaneous abortion (>3 sequential pregnancy losses); chronic hypertension; plural gestations; major congenital anomalies; infant death; or seriously ill infant. The final case status of all eligible and consenting subjects was determined using clinical information collected through extensive telephone interview and chart review. Buccal samples were self-collected and mailed by the study subjects using methods of collection, storage, and packaging that would maximize DNA yields from cytobrushes [17].

**Figure 1:**
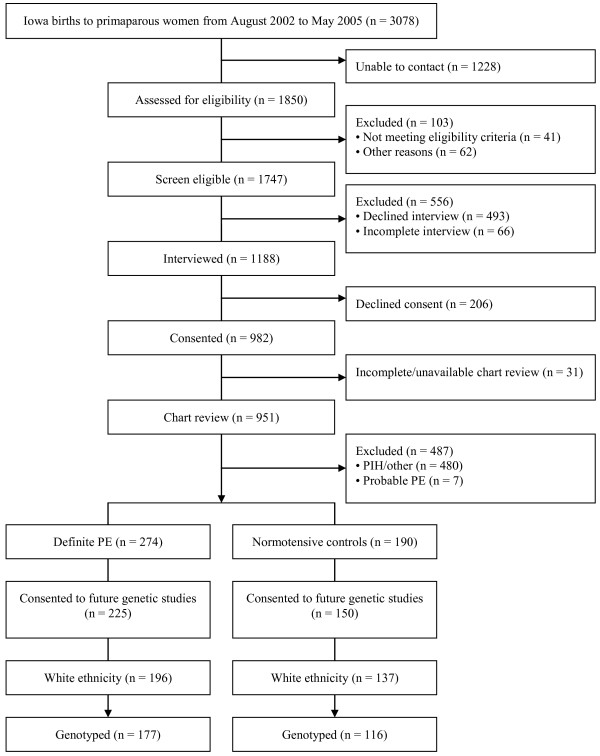
Flow chart of the SOPHIA study subject recruitment process.

Figure 1 summarizes the subject selection process. Based on information from interview and chart review, 274 PE cases and 190 normotensive controls were ascertained. The final number of cases and controls, who additionally consented to future genetic studies, was 225 and 150, respectively. Due to concerns about population stratification and the small numbers of subjects of other races/ethnicities, only white females were included in the present study (n = 196 cases and 137 controls). Among these subjects, a total of 177 cases (mean age: 27.53 ± 5.01 years) and 116 controls (mean age: 27.54 ± 5.17 years) had sufficient DNA for genome-wide SNP genotyping.

### Phenotype definition

PE was defined according to National Heart, Lung and Blood Institute (NHLBI) guidelines as having *de novo* hypertension (systolic blood pressure ≥140 mmHg or diastolic blood pressure ≥90 mmHg on two or more occasions at least six hours apart after the 20th week of gestation) and accompanying proteinuria (urine protein concentration ≥300 mg/L, equivalent to dipstick protein test value of 1+ from two or more specimens collected at least four hours apart; one or more urinary dipstick values of 2+ near the end of pregnancy; one or more catheterized dipstick value of 1+ during delivery hospitalization; or a 24-hour urine collection with protein >300 mg). Potential cases were excluded if pre-existing hypertension could not be ruled out, only partial criteria for PE were met, or a definitive diagnosis could not be made due to incomplete information. Potential controls were excluded if their medical records included any indication of high blood pressure (systolic blood pressure ≥140 mmHg or diastolic blood pressure ≥90 mmHg) in the prenatal or postpartum period, two or more high blood pressure readings in the intrapartum period, or any indication of proteinuria during pregnancy (1+ on dipstick protein testing on two or more occasions).

### GWAS genotyping

Buccal cell DNA was extracted from cytobrush samples using Puregene DNA Tissue Kits (Gentra Systems, Minneapolis, MN) following the manufacturer’s protocol, with minor modifications [17]. After extraction, DNA samples were assessed for quality by running on a 1% agarose gel. Genotyping was performed at the Rockefeller University Genomics Resource Center using Affymetrix Genome-Wide Human SNP Array 6.0 (Affymetrix, Santa Clara, CA) according to the manufacturer’s recommended protocol.

Sample quality was assessed using Dynamic Model algorithm and genotyping calls were generated using Birdseed algorithm in Genotyping Console 4.0 (Affymetrix). Samples with a quality control (QC) call rate (based on a subset of 3022 SNP markers) less than the default threshold of 86% were excluded (n = 1). This recommended QC call rate threshold is well correlated with Birdseed call rate and concordance (>99.5%) based on HapMap data (Affymetrix, 2012; personal communication). The mean QC call rate across the remaining samples (n = 292) was 94.4%.

### SNP analysis

Mitochondrial SNPs, SNPs that were monomorphic or contained only heterozygotes, SNPs that significantly deviated from Hardy-Weinberg equilibrium, or SNPs with call rates less than 95% among cases or controls were deemed to have failed QC and excluded (see Additional file 1: Table S 1 for SNP genotyping data quality summary).

Individual SNPs were tested for both allelic and genotypic associations by calculating Fisher’s exact *p*-values and using a strict Bonferroni-corrected 0.05 genome-wide significance threshold of 7.1 × 10^-8^ (α = 0.05/705,969). Presence of residual population stratification was assessed by performing a principal components analysis using the EIGENSTRAT method in EIGENSOFT version 3.0 (http://genepath.med.harvard.edu/~reich/Software.htm) [18].

### CNV detection

**Figure 2:**
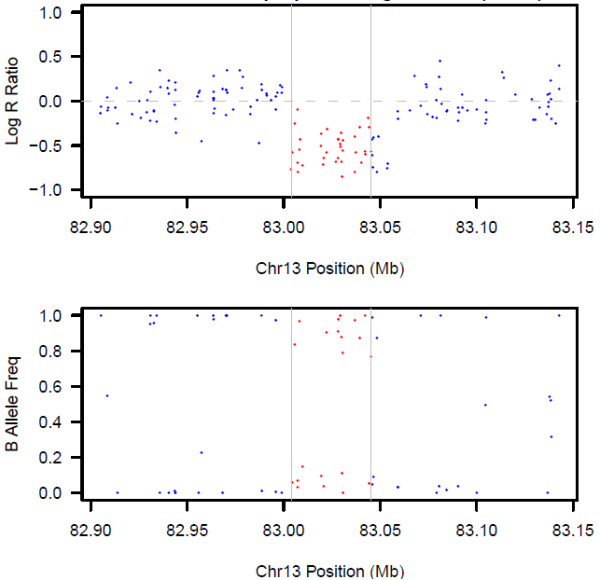
**Representative LRR and BAF plots for a genomic region called and assay confirmed as a deletion.** LRR and BAF values for each probe are represented as dots. The vertical grey bars delineate the boundaries of the algorithm-detected deletion (chr13:83.004-83.045 Mb). LRR values for the SNP and copy-number probes in the deletion (red dots) drop to the -0.5 region and BAF values for the SNP probes cluster randomly around 0 or 1. In comparison, the flanking normal chromosomal regions have LRR values centered around zero with three BAF clusters (blue dots).

Four algorithms served to identify CNVs from the genome-wide SNP data. Three algorithms, Birdseye [19], PennCNV June 16, 2011 version [20], and QuantiSNP version 2.3 beta [21], implement a hidden Markov model that integrates multiple sources of information, including log R ratio (LRR; a measure of total signal intensity of probes) and B allele frequency (BAF; a measure of relative intensity ratio of allelic probes), to infer CNV calls for individual genotyped samples. An example of LRR and BAF plots for a region called and confirmed as a deletion is shown in Figure 2. The last algorithm, Canary [19], utilizes a one-dimensional Gaussian mixture model to detect common CNVs. Birdseye and Canary were run as part of the Birdsuite version 1.5.5 toolset. The unified output of Birdseye and Canary was used for further analysis and these two algorithms are hereafter referred to as one algorithm, Birdsuite. Detection algorithms were run under default settings for Affymetrix SNP 6.0 microarray using LRR and/or BAF data from all SNP and copy-number probes in all 293 genotyped subjects. All samples were processed in one batch. The presence of CNVs on the X chromosome is of interest as epigenetic investigation found a link between X chromosome inactivation and PE among white females [22]. Thus, both autosomal and X chromosomes were analyzed.

A large number of algorithm calls may be indicative of low sample quality [23]. To assess sample quality, the right-skewed distribution of sample calls was first log-transformed for each algorithm. A sample was considered to have failed QC and removed from analysis (n = 8 cases and 2 controls) if it had an extremely large number of CNV calls, defined as having a value greater than three standard deviations from the mean of the log-transformed number of CNV calls per sample for at least one of the algorithms.

A modified CNVision program [24] was used to merge, analyze, and annotate the outputs of Birdsuite, PennCNV, and QuantiSNP. The merge function of CNVision identifies and merges CNV calls made by all algorithms that have overlap of ≥1 base pair and determines the percentage of call overlap by algorithm within this region. Merged CNV calls were excluded (n = 210,583) when at least one of the following conditions were met: 1) <50% overlap between two algorithms and <25% overlap among three algorithms; 2) less than ten consecutive SNP or copy-number probes; or 3) both deletion and amplification calls made by different algorithms in the same genomic region in a given sample.

### CNV prioritization

An objective prioritization strategy was employed to generate a list of candidate CNV regions most enriched among cases. Odds ratios (ORs) were calculated for this purpose. Therefore, CNVs with ORs ≥ 2.5, comparing PE cases and normotensive controls, and CNVs called in ≥3 cases and absent in controls were selected for additional consideration. Recurrent regions overlapping centromeric and telomeric regions according to PennCNV definitions or containing less than five consecutive microarray probes were excluded. The normal expected frequency of these shortlisted CNVs was assessed in an independent comparison group of white female controls (n = 774) genotyped using Affymetrix SNP 6.0 microarrays from a GWAS of schizophrenia (NCBI study accession: phs000021.v3.p2) [25]. As the schizophrenia study controls were not screened with the same criteria as SOPHIA controls, it was expected that 2-7% of these women who become pregnant would develop PE. However, assuming a positive relationship exists, this selection bias may in fact attenuate the association, providing a conservative estimate of risk for the prioritization process. CNVs were detected in the same manner as the PE cases and controls; however, samples were batch-processed by 96-well plate. The same sample QC and merged CNV call criteria were applied. Regions were further considered when ORs comparing PE cases and schizophrenia study controls were positive with their 95% confidence intervals excluding the null value (OR = 1) or when calls were absent among controls.

As DNA quantities were very limited, it was decided *a priori* that only copy-number deletions would be selected for assay confirmation due to their generally more deleterious nature relative to amplifications [26]. Regions to be assayed in the entire case-control dataset using quantitative real-time PCR (RT-qPCR) were selected based on the presence of genes at or near (≤100 kb) the CNV, the availability of DNA for samples displaying the CNV, and having the majority of samples without the deletion be copy normal. LRR and BAF plots were also visually inspected for a clear change in probe hybridization intensity and zygosity, respectively, to ensure patterns consistent with calls.

### Copy-number genotyping

Copy-number genotyping was performed using TaqMan Copy Number Assays (Applied Biosystems, Foster City, CA) following the manufacturer’s suggested protocol. All reactions were performed in triplicate with 5 ng of sample DNA for each reaction. The copy-number assay detects the target genomic region and consists of a FAM dye-labeled minor groove binder probe and unlabeled PCR primers. This target assay was amplified simultaneously with a genomic reference assay, which includes a VIC dye-labeled TAMRA probe and primers, in a duplex RT-qPCR. The reference assay detects the RNaseP gene, a known two copy region in the diploid genome. Plates were run using the Bio-Rad CFX384 machine (Bio-Rad Laboratories, Hercules, CA) under recommended PCR cycling conditions (95°C for 10 minutes followed by 40 cycles of 95°C for 15 seconds and 60°C for 1 minute). Cycle thresholds (*C*_T_) were calculated using CFX Manager Software version 2.0 (Bio-Rad) and reformatted with a manual *C*_T_ threshold specification of 0.20 for import into CopyCaller Software version 1.0 (Applied Biosystems). Wells with VIC *C*_T_ values exceeding the default filtering threshold of 32 were excluded. Relative quantification analysis was performed with CopyCaller software where discrete copy-number classes were determined by employing a maximum-likelihood algorithm on the real-time data.

## Results

Genomic positions are designated according to NCBI36/hg18 human genome assembly.

### SNP associations

A total of 292 of the 293 genotyped samples (177 cases and 115 controls) passed the default QC call rate threshold (≥86%), with overall sample call rates ranging from 86.1-99.3%. EIGENSTRAT analysis showed no evidence of population stratification (*p* > 0.15 for first 10 principal components).

No SNP surpassed the Bonferroni-corrected significance threshold of 7.1 × 10^-8^ for the Fisher’s exact allelic or genotypic tests. The top four SNP candidates had an allelic or genotypic *p*-value between 10^-5^ and 10^-6^ (Table 1).

**Table 1:** **Association (*p*-value) of the top four SNP candidates with PE**^***a***^

**SNP**^***b***^	**Minor allele frequency (%)**		**Fisher’s exact** ***p*-value**		**Chr.**	**Position**	**SNP type**	**Closest gene**	**Distance to gene (kb)**
	**Cases**	**Controls**	**Allelic**	**Genotypic**					
	**(n = 177)**	**(n = 115)**							
rs1426409	22.6	31.1	2.58 × 10^-2^	3.14 × 10^-6^	4	36850339	Intergenic	*KIAA1239*	72.7
rs17686866	14.2	29.1	1.97 × 10^-5^	3.80 × 10^-6^	1	215251888	Intronic	*ESRRG*	0
rs9831647	38.1	48.3	1.65 × 10^-2^	9.36 × 10^-6^	3	8546110	Intronic	*LMCD1*	0
rs10743565	24.3	42.8	5.00 × 10^-6^	1.64 × 10^-5^	12	25551846	Intronic	*IFLTD1*	0

^*a*^SNPs with allelic or genotypic *p*-value between 10^-5^ and 10^-6^.

^*b*^Ordered according to smallest to largest allelic or genotypic Fisher’s exact *p*-values.

### CNV detection and confirmation

A total of 14,181 autosomal CNVs, 9074 deletions and 5107 amplifications meeting inclusion criteria, were detected among the 169 case and 114 control subjects that passed the sample QC. The identified variants ranged in size from 241 base pairs to nearly 4.1 Mb (median = 22.8 kb). The copy-number deletions were merged into 2770 regions, as defined by the minimum region of overlap across sample calls. Among these, three merged regions of recurrent deletions that were detected in multiple cases, but were less common or undetected in controls, met the pre-specified screening criteria (Table 2; see Additional file 2: Table S 2 for an annotated list of deleted regions in autosomal chromosomes meeting initial prioritization criteria). The exact breakpoints for these three candidate CNVs remain undetermined, but microarray data suggest that these breakpoints may vary in subjects harboring the deletions. Correspondingly, copy-number amplifications were merged into 2981 regions with 21 regions meeting initial prioritization criteria (Additional file 3: Table S 3). For X chromosome, 107 deletion and 114 amplification calls were included and merged into 97 regions of deletion and 97 regions of amplification. Only five merged regions of recurrent amplifications in X chromosome were found to be enriched in cases (Additional file 4: Table S 4). These minimal common regions of amplification are interesting candidates for further investigation.

**Table 2:** Recurrent copy-number deletions identified in PE cases and controls

**CNV region**^***a***^	**Gene contents**	**Copy-number frequency**^***b***^		
		**Cases**^***c***^	**Controls**^***c***^	**Population-based controls**^***d***^
Chr13:83.004-83.045 Mb	Intergenic	5/169	0/114	6/770
Chr16:14.972-14.987 Mb	*PDXDC1*	8/169	1/114	14/770
Chr19:48.461-48.476 Mb	*PSG11*	5/169	1/114	2/770

^*a*^Minimum region of overlap across all subjects harboring the deletion.

^*b*^CNV frequencies are derived from SNP microarray data.

^*c*^Algorithm detected CNVs were all validated by RT-qPCR with the exception of one case in each of the chromosome 13 and 16 regions due to unavailable DNA. Furthermore, RT-qPCR identified a heterozygous deletion in one case and two control subjects in the chromosome 16 CNV region as well as a case subject in the chromosome 19 region that were called as copy normal by the algorithms.

^*d*^White female subjects from a GWAS of schizophrenia genotyped using Affymetrix SNP 6.0 microarrays. CNVs were detected using the same algorithms as the PE GWAS. Four population-based controls did not pass sample QC.

The most enriched deletion among cases is ~15 kb in length and was detected in eight cases (4.7%) and one control (0.9%). The shared region of overlap for this deletion in 16p13.11 extends from 14.972 to 14.987 Mb and encompasses the *PDXDC1* gene. This deletion was identified in 14 of the 770 (1.8%) schizophrenia study controls that passed QC. The 41 kb intergenic deletion at 13q31.1 was detected in five cases (3.0%) and zero controls with a shared region from 83.004 to 83.045 Mb. This deletion was also identified in six of the 770 (0.8%) schizophrenia study controls. The nearest gene, *SLITRK1*, is located 304.17 kb downstream of this region. The third CNV overlaps with the *PSG11* gene (alternatively spliced as *PSG9* and *PSG11s*) in 19q13.31 from 48.461 to 48.476 Mb. This deletion was detected in five cases (3.0%), one control (0.9%), and only two schizophrenia study controls (0.3%). It was also reported in very low frequencies by three studies [27-29] listed in the Database of Genomic Variants (http://projects.tcag.ca/variation/) [30]. One study reported this deletion in 2/1854 (0.1%) controls, one in 1/776 (0.1%) controls, and the last in 11/2026 (0.5%) controls.

All samples with available DNA were genotyped using pre-designed TaqMan assays for the deletions in 13q31.1 (ABI assay ID: Hs03297694_cn) and 16p13.11 (Hs03938043_cn). The deletion in 19q13.31 was genotyped using a custom assay with a target region of chr19:48,461,720-48,462,020 designed using the Copy Number Assay Workflow Builder (http://www5.appliedbiosystems.com/tools/cnv/). Two cases failed to amplify across all three genomic regions while an additional case failed for the chromosome 19 region. These three samples were algorithm called as copy normal in the three regions of interest. No samples were excluded for surpassing the VIC *C*_T_ value threshold.

No false-positive and only a few false-negative algorithm calls were found by laboratory verification. The presence of all putative deletions called by the algorithms in these three genomic regions was successfully confirmed by RT-qPCR, except one case subject in each of the chromosome 13 and 16 CNV regions where DNA was unavailable. The RT-qPCR assay showed that a heterozygous copy-number deletion was present in 4/155 cases and none in 98 controls in the 13q31.1 region and 8/155 cases and 3/98 controls in the 16p13.11 region, including deletions detected in one case and two controls that were not algorithm called. For the deletion in chromosome 19, a heterozygous deletion was confirmed in five cases and one control with an additional case deletion detected that was called copy normal by the CNV calling algorithms.

## Discussion

Genome-wide analysis of CNVs identified three rare deletions enriched in PE, two of which disrupt genes, confirmed by laboratory validation. Although copy-number amplifications were not selected for assay, several candidate regions were detected in the autosomal and X chromosomes. The most interesting deletion, based on possible biological pathways, is the 15 kb deletion in 19q13.31 that encompasses the *PSG11* gene. Pregnancy-specific glycoproteins (PSGs) are mainly produced by placental syncytiotrophoblasts during pregnancy and constitute a subgroup of the carcinoembryonic antigen family, which belongs to the immunoglobulin superfamily [31]. Studies have shown that several members of the PSG gene family, including PSG11, induce dose-dependent monocytic secretion of anti-inflammatory cytokines, which physiologically contribute to the maintenance of a successful pregnancy. In contrast, activation of coagulation mechanisms by pro-inflammatory cytokines can lead to maternal endothelial dysfunction, vasculitis, and consequently uteroplacental hypoxia [32]. Hypoxia plays a crucial role in placental pathologies such as PE. Inadequate uteroplacental oxygenation is believed to be involved in molecular events leading to the clinical manifestations of PE [33].

**Figure 3:**
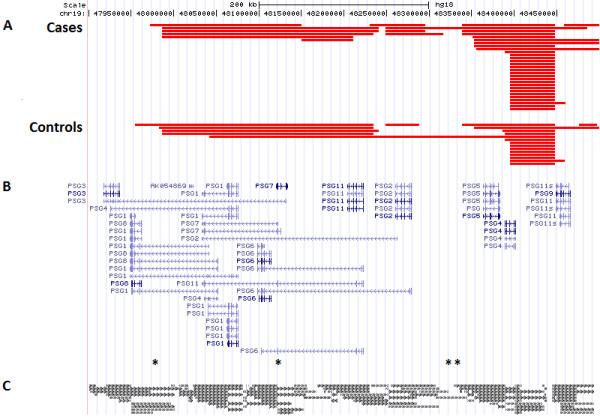
**UCSC Genome Browser plot of the PSG gene family region (chr19:47.918-48.465 Mb).** (**A**) Each red horizontal bar represents the length and breakpoints of a putative deletion called in PE cases or controls. (**B**) UCSC Genes located within this region. Asterisks indicate the genomic positions of nominally significant SNPs (from left to right: rs4030933, rs2159027, rs10417319, and rs10402173). (**C**) Segmental duplications of ≥1 kb with 90-98% sequence similarity.

**Figure 4:**
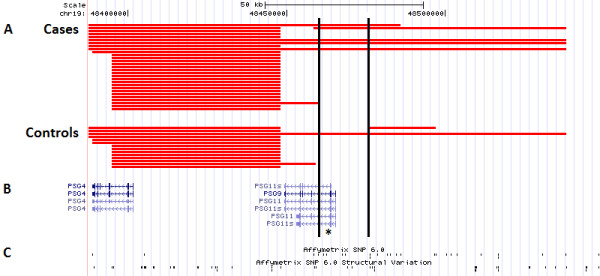
**UCSC Genome Browser plot of the copy-number deletion at chr19:48.461-48.476 Mb.** (**A**) The vertical black lines indicate the minimum region of overlap across all subjects harboring the deletion. Each red horizontal bar represents the length and breakpoints of a deletion detected in either PE cases or controls. Exact CNV breakpoints are unknown. (**B**) UCSC Genes located within this region. The asterisk denotes the target region (chr19:48,461,720-48,462,020) of the custom TaqMan copy-number assay. (**C**) Genomic positions of SNP and structural variation copy-number probes used in the Affymetrix SNP 6.0 microarray.

*PSG11* is located at the telomeric end of the PSG gene family cluster (chr19:47.918-48.465 Mb) and is arranged in tandem with the other PSG genes [34]. This cluster has a high density of segmental duplications (low copy repeats). CNVs are not uniformly distributed in the human genome, but tend to be enriched in regions of segmental duplication. Segmental duplications predispose affected regions to recurrent chromosomal rearrangements through non-allelic homologous recombination and may be the underlying mechanism in the formation of CNVs within this cluster (see Figure 3 for locations of putative segmental duplications within the PSG gene family cluster) [35,36]. According to the microarray data, there is a highly variable genomic region upstream of the deletion of interest in 19q13.31 (Figure 4). It is apparent that the 3’ boundary of this upstream CNV hotspot terminates before the alternatively spliced *PSG11* region. However, the enriched minimum deleted region appears to be primarily an extension of this more common upstream deleted region (5/6 deletions). Although the exact breakpoints remain undetermined, RT-qPCR confirmed the deletion breakpoints to be varying in this genomic locus; the minimal deleted region was not detected in every sample with the upstream deletion. There is also no evidence of appreciable enrichment in the minimum region of overlap (48.396-48.448 Mb) for upstream deletion, which was detected in 29 cases (17.2%) and 14 controls (12.3%). Furthermore, inspection of the entire PSG gene family region (chr19:47.918-48.465 Mb) suggests that only the deletion in the 48.461 to 48.476 Mb region, which disrupts *PSG11*, is enriched in PE cases compared to controls (Figure 3).

Studies have shown that CNVs can lead to diseases or other phenotypes by various mechanisms, including the disruption of functional genes [37]. Therefore, deletions are under strong purifying selection and are preferentially located outside of genes and highly conserved elements in the genome [15]. The rarity of the deletion in the functionally relevant *PSG11* gene, within a hotspot of genomic instability, suggests that there is selective pressure acting against this CNV. Consequently, although *PSG11* and its alternatively spliced variants have yet to be directly linked to PE, they represent intriguing candidates for future research.

The validity of CNVs identified in array-based studies is algorithm-dependent. High variability in findings exists among CNV detection methods, along with substantial false-positive and false-negative rates [38]. In an attempt to increase the accuracy of CNV prediction, this study employed three algorithms (Birdsuite (Birdseye and Canary), PennCNV, and QuantiSNP) with stringent overlapping criteria to call CNVs on a genome-wide level. RT-qPCR confirmed 4/4 predicted deletions in 13q31.1 with DNA available for assay. All calls in this region were in 100% agreement by the three algorithms, with no false-positive or false-negative calls. For the deletions in 16p13.11 and 19q13.31, all calls in cases and controls with available DNA were confirmed by assay. However, there were false-negative calls in one case and two controls for the former and in one case subject for the latter CNV. In both of these deletions, the minimum region of overlap among cases and controls was called by at least two algorithms. Visual inspection of signal intensity plots for the assay-confirmed CNV calls supported the heterozygous copy-number deletion call, where the LRR drops to the -0.5 region and the BAF clusters around 0 or 1. Although the plots revealed that LRR and BAF patterns were consistent with a deletion, the false-negative calls appear to be caused by a high signal-to-noise ratio in the array data at these regions. Despite the potential exclusion of candidate CNV regions due to the stringent CNV detection method applied in this study, it was successful in reducing the false-positive and false-negative rates that are common in studies that rely on array-based technologies to infer CNVs.

The present GWAS did not identify any variants that were associated with PE at a genome-wide level of significance. The lack of significant findings may be due to the insufficient statistical power of this study to detect variants of small or moderate effect size, increasing the false-negative rate. Furthermore, forcing raw measurements with continuous distributions (i.e. probe intensity measurements) into discrete copy-number classes (e.g. gain, no change, loss) when calling CNVs using array-based technologies may result in the loss of substantial statistical power [39]. Discovery of disease-associated variants may be biased towards genomic regions with better coverage by the SNP microarray used in the study. Study findings also assumed that genotype does not influence subject selection. Although a reasonable assumption, there may be situations where the genotype affects participation rates through its association with certain selection factors [40].

Although no SNPs reached genome-wide significance, it is interesting to note that four SNPs within the PSG gene family cluster (chr19:47.918-48.465 Mb) and immediate flanking regions (±10 kb) reached nominal significance, including one in the intronic region of *PSG134*, and *8* genes, one in the intronic region of *PSG2367*, and *11* genes, and two located intergenically between *PSG2* and *PSG5* (see Figure 3 for the SNP positions within the gene cluster). These nominally significant SNPs may be functionally important; regulatory elements, such as enhancers and repressors, may reside in intronic regions or up- and downstream of the transcriptional unit [41]. Further replication and functional studies are warranted to elucidate the roles of these putative risk variants within the PSG gene family region in PE.

## Conclusions

Genome-wide CNV analysis discovered three rare but recurrent deletions that may confer risk for PE, including a potentially functionally important copy-number deletion in the *PSG11* gene. Larger replication studies are needed to confirm these findings. Although no significant SNPs were discovered, the list of top SNP candidates generated by the present study may be a useful basis for future genetic association studies of PE.

## Competing interests

The authors declare that they have no competing interests, financial or otherwise.

## Authors’ contribution

ATD, JH, and EWT conceived and designed the study. AFS and EWT recruited subjects and collected DNA samples. LZ performed the statistical analysis. MBB, ATD, KMW, and LZ interpreted the results. LZ drafted the manuscript. All authors revised for important intellectual content, read, and approved the final manuscript.

## Pre-publication history

The pre-publication history for this paper can be accessed here:

http://www.biomedcentral.com/1471-2393/12/61/prepub

## Supplementary Material

Additional file 1 Table S1.SNP genotyping data quality. SNP genotyping data quality summary. Click here for file

Additional file 2 Table S2.Regions of autosomal copy-number deletion meeting initial prioritization criteria. Annotated list of autosomal deletions enriched among cases that met initial prioritization criteria. Click here for file

Additional file 3 Table S3.Regions of autosomal copy-number amplification meeting initial prioritization criteria. Annotated list of autosomal amplifications enriched among cases that met initial prioritization criteria. Click here for file

Additional file 4 Table S4.X chromosome CNV regions meeting initial prioritization criteria. Annotated list of candidate CNV regions in X chromosome enriched among cases that met initial prioritization criteria. Click here for file
