# Supplementary material for: Genome-wide association study identifies a maternal copy-number deletion in PSG11 enriched among preeclampsia patients
Source: BMC Pregnancy Childbirth. 2012 Jun 29;12:61. doi: 10.1186/1471-2393-12-61 (PMC3476390; doi:10.1186/1471-2393-12-61)
Supplement: Additional file 1 — Table S1.SNP genotyping data quality. SNP genotyping data quality summary. [file 1471-2393-12-61-S1.doc]

**Table S1.** SNP genotyping data quality

| Total number of SNPs | 909,622 |
| --- | --- |
|  |  |
| Number of mitochondrial SNPs | 1591 |
| Number of SNPs with call rate < 95% | 162,573 |
| Number of SNPs with no polymorphism or only heterozygotes observed | 37,449 |
| Number of SNPs with HWE *χ^2^* > 50 | 2040 |
|  |  |
| Final number of SNPs | 705,969 |
