# Supplementary material for: Genome-wide association study identifies a maternal copy-number deletion in PSG11 enriched among preeclampsia patients
Source: BMC Pregnancy Childbirth. 2012 Jun 29;12:61. doi: 10.1186/1471-2393-12-61 (PMC3476390; doi:10.1186/1471-2393-12-61)
Supplement: Additional file 2 — Table S2.Regions of autosomal copy-number deletion meeting initial prioritization criteria. Annotated list of autosomal deletions enriched among cases that met initial prioritization criteria. [file 1471-2393-12-61-S2.doc]

**Table S2.** Regions of autosomal copy-number deletion meeting initial prioritization criteria

| **CNV region**  **(chromosome: start position-stop position)** | **Region size** | **# probes within region** | **# PE case deletion calls (%)**  **(n = 169)** | **# PE control deletion calls (%)**  **(n = 114)** | **OR (95% CI) *^a^*** | **F-exact *p*-value** | **# schizophrenia study control deletion calls (%)**  **(n = 770)** | **OR (95% CI) *^b^*** | **# genes (exons) in region** | **Gene in region** | **Location** | **5' gene** | **5' distance**  **(kb)** | **3' gene** | **3' distance**  **(kb)** |
| --- | --- | --- | --- | --- | --- | --- | --- | --- | --- | --- | --- | --- | --- | --- | --- |
|  |  |  |  |  |  |  |  |  |  |  |  |  |  |  |  |
| chr13:83003850-83045171 | 41321 | 40 | 5 (2.96) | 0 (0) | - | 0.08 | 6 (0.78) | 3.88 (1.17-12.87) | 0 (0) | - | Intergenic | *SPRY2* | 3190.76 | *SLITRK1* | 304.17 |
| chr13:83045171-83054013 | 8842 | 9 | 6 (3.55) | 0 (0) | - | 0.08 | 6 (0.78) | 4.69 (1.49-14.72) | 0 (0) | - | Intergenic | *SPRY2* | 3232.08 | *SLITRK1* | 295.33 |
| chr16:14971218-14987956 | 16738 | 10 | 8 (4.73) | 1 (0.88) | 5.61 (0.69-45.52) | 0.09 | 14 (1.82) | 2.68 (1.11-6.50) | 1 (1) | *PDXDC1* | Exon | in | 0 | in | 0 |
| chr16:14956269-14957386 | 1117 | 9 | 7 (4.14) | 1 (0.88) | 4.88 (0.59-40.24) | 0.15 | 12 (1.56) | 2.73 (1.06-7.04) | 0 (0) | - | Intergenic | *NPIP* | 2.84 | *PDXDC1* | 18.95 |
| chr16:14965859-14971218 | 5359 | 12 | 8 (4.73) | 2 (1.75) | 2.78 (0.58-13.35) | 0.33 | 13 (1.69) | 2.89 (1.18-7.10) | 0 (0) | - | Intergenic | *NPIP* | 12.43 | *PDXDC1* | 5.12 |
| chr10:47011195-47122809 | 111614 | 113 | 4 (2.37) | 0 (0) | - | 0.15 | 2 (0.26) | 9.31 (1.69-51.25) | 0 (0) | - | Intergenic | *LOC642826* | 347.70 | *ANTXRL* | 5.43 |
| chr10:47122809-47165567 | 42758 | 64 | 3 (1.78) | 0 (0) | - | 0.28 | 2 (0.26) | 6.94 (1.15-41.86) | 1 (18) | *ANTXRL* | Exon | in | 0 | In | 0 |
| chr1:143742309-143785910 | 43601 | 15 | 3 (1.78) | 0 (0) | - | 0.28 | 0 (0) | - | 1 (0) | *PDE4DIP* | Intron | *PDE4DIP* | 9.79 | *PDE4DIP* | 1.08 |
| chr6:77073620-77084501 | 10881 | 34 | 3 (1.78) | 0 (0) | - | 0.28 | 1 (0.13) | 13.90 (1.44-134.44) | 0 (0) | - | Intergenic | *IMPG1* | 234.57 | *HTR1B* | 1144.17 |
| chr7:109214127-109220983 | 6856 | 12 | 3 (1.78) | 0 (0) | - | 0.28 | 0 (0) | - | 0 (0) | - | Intergenic | *C7orf66* | 902.25 | *EIF3IP1* | 165.54 |
| chr7:141891589-141909210 | 17621 | 19 | 3 (1.78) | 0 (0) | - | 0.28 | 1 (0.13) | 13.90 (1.44-134.44) | 0 (0) | - | Intergenic | *TRYX3* | 287.23 | *PRSS1* | 227.68 |
| chr7:97240411-97242449 | 2038 | 6 | 3 (1.78) | 0 (0) | - | 0.28 | 0 (0) | - | 0 (0) | - | Intergenic | *TAC1* | 32.69 | *ASNS* | 76.93 |
| chr19:48460022-48476132 | 16110 | 8 | 5 (2.96) | 1 (0.88) | 3.45 (0.40-29.89) | 0.41 | 2 (0.26) | 11.71 (2.25-60.87) | 1 (2) | *PSG11* | Exon | in | 0 | In | 0 |
| chr19:48497168-48538234 | 41066 | 22 | 4 (2.37) | 1 (0.88) | 2.74 (0.30-24.83) | 0.65 | 2 (0.26) | 9.31 (1.69-51.25) | 0 (0) | - | Intergenic | *PSG11* | 31.65 | *PRG1* | 6.81 |
|  |  |  |  |  |  |  |  |  |  |  |  |  |  |  |  |

*^a^* OR comparing PE cases and normotensive controls

*^b^* OR comparing PE cases and schizophrenia study controls

Candidate CNV regions that met initial prioritization criteria were further prioritized for assay on the entire case-control dataset using real-time quantitative PCR based on the presence of genes at or near (≤ 100 kb) the CNV, the availability of DNA for samples displaying the CNV, having the majority of samples without the deletion be copy normal, and visual inspection of log R ratio and B allele frequency plots to ensure patterns consistent with calls. The merged CNV regions selected for targeted genotyping are highlighted in yellow. Black box borders denote contiguous merged CNV regions. Genomic positions are designated according to NCBI36/hg18 human genome assembly. Abbreviations: CI, confidence interval; CNV, copy-number variant; OR, odds ratio; PE, preeclampsia.
