# Supplementary material for: Genome-wide association study identifies a maternal copy-number deletion in PSG11 enriched among preeclampsia patients
Source: BMC Pregnancy Childbirth. 2012 Jun 29;12:61. doi: 10.1186/1471-2393-12-61 (PMC3476390; doi:10.1186/1471-2393-12-61)
Supplement: Additional file 3 — Table S3.Regions of autosomal copy-number amplification meeting initial prioritization criteria. Annotated list of autosomal amplifications enriched among cases that met initial prioritization criteria. [file 1471-2393-12-61-S3.doc]

**Table S3.** Regions of autosomal copy-number amplification meeting initial prioritization criteria

| **CNV region**  **(chromosome: start position-stop position)** | **Region size** | **# probes within region** | **# PE case deletion calls (%)**  **(n = 169)** | **# PE control deletion calls (%)**  **(n = 114)** | **OR (95% CI) *^a^*** | **F-exact *p*-value** | **# schizophrenia study control deletion calls (%)**  **(n = 770)** | **OR (95% CI) *^b^*** | **# genes (exons) in region** | **Gene in region** | **Location** | **5' gene** | **5' distance (kb)** | **3' gene** | **3' distance (kb)** |
| --- | --- | --- | --- | --- | --- | --- | --- | --- | --- | --- | --- | --- | --- | --- | --- |
| chr8:39507456-39508281 | 826 | 5 | 10 (5.92) | 1 (0.88) | 7.11 (0.90-56.31) | 0.05 | 21 (2.73) | 2.24 (1.04-4.86) | 0 (0) | - | Intergenic | *ADAM3A* | 7.83 | *ADAM18* | 53.02 |
| chr4:115393012-115393105 | 94 | 5 | 4 (2.37) | 0 (0) | - | 0.15 | 2 (0.26) | 9.31 (1.69-51.25) | 0 (0) | - | Intergenic | *ARSJ* | 272.69 | *UGT8* | 345.95 |
| chr7:76037411-76126690 | 89280 | 39 | 4 (2.37) | 0 (0) | - | 0.15 | 3 (0.39) | 6.20 (1.37-27.96) | 1 (7) | *POMZP3* | Exon | In | 0 | In | 0 |
| chr7:61835137-61889916 | 54780 | 9 | 6 (3.55) | 1 (0.88) | 4.16 (0.49-35.02) | 0.25 | 4 (0.52) | 7.05 (1.97-25.26) | 0 (0) | - | Intergenic | *ZNF479* | 4623.62 | *LOC643955* | 499.19 |
| chr7:61463516-61624608 | 161093 | 25 | 3 (1.78) | 0 (0) | - | 0.28 | 0 (0) | - | 0 (0) | - | Intergenic | *ZNF479* | 4252.00 | *LOC643955* | 764.50 |
| chr7:61889916-61933674 | 43759 | 7 | 4 (2.37) | 1 (0.88) | 2.74 (0.30-24.83) | 0.65 | 4 (0.52) | 4.64 (1.15-18.75) | 0 (0) | - | Intergenic | *ZNF479* | 4678.40 | *LOC643955* | 455.43 |
| chr5:5732779-5734033 | 1255 | 25 | 3 (1.78) | 0 (0) | - | 0.28 | 1 (0.13) | 13.90 (1.44-134.44) | 0 (0) | - | Intergenic | *KIAA0947* | 189.44 | *FLJ33360* | 629.52 |
| chr9:40519803-40552022 | 32220 | 7 | 3 (1.78) | 0 (0) | - | 0.28 | 2 (0.26) | 6.94 (1.15-41.86) | 0 (0) | - | Intergenic | *FAM74A1* | 622.56 | *FAM75A3* | 138.27 |
| chr10:45926794-46005056 | 78263 | 6 | 3 (1.78) | 0 (0) | - | 0.28 | 2 (0.26) | 6.94 (1.15-41.86) | 2 (10) | *PTPN20A, PTPN20B* | Exon | In | 0 | In | 0 |
| chr12:118475003-118478901 | 3899 | 21 | 3 (1.78) | 0 (0) | - | 0.28 | 0 (0) | - | 0 (0) | - | Intergenic | *CCDC60* | 11.77 | *LOC387890* | 36.75 |
| chr13:27559806-27561307 | 1502 | 15 | 3 (1.78) | 0 (0) | - | 0.28 | 0 (0) | - | 1 (0) | *FLT3* | Intron | *FLT3* | 17.06 | *FLT3* | 11.30 |
| chr17:692301-715637 | 23337 | 14 | 3 (1.78) | 0 (0) | - | 0.28 | 1 (0.13) | 13.90 (1.44-134.44) | 1 (0) | *NXN* | Intron | *NXN* | 16.23 | *NXN* | 113.67 |
| chr17:715637-722158 | 6522 | 7 | 3 (1.78) | 0 (0) | - | 0.28 | 0 (0) | - | 1 (0) | *NXN* | Intron | *NXN* | 39.57 | *NXN* | 107.15 |
| chr19:32439833-32573026 | 133194 | 18 | 3 (1.78) | 0 (0) | - | 0.28 | 0 (0) | - | 0 (0) | - | Intergenic | *LOC100101266* | 8301.74 | *LOC148189* | 400.22 |
| chr3:131291501-131294045 | 2545 | 6 | 8 (4.73) | 2 (1.75) | 2.78 (0.58-13.35) | 0.33 | 13 (1.69) | 2.89 (1.18-7.10) | 1 (2) | *LOC644974* | Exon | In | 0 | In | 0 |
| chr9:69267237-69267842 | 606 | 10 | 5 (2.96) | 1 (0.88) | 3.45 (0.40-29.89) | 0.41 | 3 (0.39) | 7.79 (1.84-32.94) | 0 (0) | - | Intergenic | *LOC100133920* | 312.47 | *FOXD4L5* | 197.68 |
| chr9:69247488-69267237 | 19750 | 8 | 4 (2.37) | 1 (0.88) | 2.74 (0.30-24.83) | 0.65 | 1 (0.13) | 18.64 (2.07-167.88) | 0 (0) | - | Intergenic | *LOC100133920* | 292.72 | *FOXD4L5* | 198.29 |
| chr11:37724389-37791537 | 67149 | 39 | 5 (2.96) | 1 (0.88) | 3.45 (0.40-29.89) | 0.41 | 4 (0.52) | 5.84 (1.55-21.98) | 0 (0) | - | Intergenic | *C11orf74* | 1087.00 | *LRRC4C* | 2300.79 |
| chr8:62544158-62549821 | 5664 | 10 | 4 (2.37) | 1 (0.88) | 2.74 (0.30-24.83) | 0.65 | 4 (0.52) | 4.64 (1.15-18.75) | 1 (0) | *RLBP1L1* | Intron | *RLBP1L1* | 10.50 | *RLBP1L1* | 24.75 |
| chr16:22465433-22612022 | 146590 | 73 | 4 (2.37) | 1 (0.88) | 2.74 (0.30-24.83) | 0.65 | 3 (0.39) | 6.20 (1.37-27.96) | 1 (9) | *LOC653786* | Exon | In | 0 | In | 0 |
| chr22:41222277-41227226 | 4950 | 5 | 4 (2.37) | 1 (0.88) | 2.74 (0.30-24.83) | 0.65 | 3 (0.39) | 6.20 (1.37-27.96) | 1 (1) | *SERHL* | Exon | In | 0 | In | 0 |

*^a^* OR comparing PE cases and normotensive controls

*^b^* OR comparing PE cases and schizophrenia study controls

Black box borders denote contiguous merged CNV regions. Genomic positions are designated according to NCBI36/hg18 human genome assembly. Abbreviations: CI, confidence interval; CNV, copy-number variant; OR, odds ratio; PE, preeclampsia.
