# Supplementary material for: Genome-wide association study identifies a maternal copy-number deletion in PSG11 enriched among preeclampsia patients
Source: BMC Pregnancy Childbirth. 2012 Jun 29;12:61. doi: 10.1186/1471-2393-12-61 (PMC3476390; doi:10.1186/1471-2393-12-61)
Supplement: Additional file 4 — Table S4.X chromosome CNV regions meeting initial prioritization criteria. Annotated list of candidate CNV regions in X chromosome enriched among cases that met initial prioritization criteria. [file 1471-2393-12-61-S4.doc]

**Table S4.** X chromosome CNV regions meeting initial prioritization criteria

| **CNV region**  **(chromosome: start position-stop position)** | **CNV type** | **Region size** | **# probes within region** | **# PE case deletion calls (%)**  **(n = 169)** | **# PE control deletion calls (%)**  **(n = 114)** | **OR (95% CI) *^a^*** | **F-exact *p*-value** | **# schizophrenia study control deletion calls (%)**  **(n = 770)** | **OR (95% CI) *^b^*** | **# genes (exons) in region** | **Gene in region** | **Location** | **5' gene** | **5' distance (kb)** | **3' gene** | **3' distance (kb)** |
| --- | --- | --- | --- | --- | --- | --- | --- | --- | --- | --- | --- | --- | --- | --- | --- | --- |
| chrX:153219802-153283427 | Amplification | 63626 | 84 | 6 (3.55) | 0 (0) | - | 0.08 | 0 (0) | - | 5 (62) | *DNASE1L1, EMD, FLNA, RPL10, SNORA70* | Exon | In | 0 | In | 0 |
| chrX:599597-734694 | Amplification | 135098 | 24 | 3 (1.78) | 0 (0) | - | 0.28 | 1 (0.13) | 13.9 (1.44-134.44) | 0 (0) | - | Intergenic | *SHOX* | 59.45 | *CRLF2* | 540.19 |
| chrX:734694-837036 | Amplification | 102343 | 15 | 3 (1.78) | 0 (0) | - | 0.28 | 2 (0.26) | 6.94 (1.15-41.86) | 0 (0) | - | Intergenic | *SHOX* | 194.55 | *CRLF2* | 437.85 |
| chrX:2965790-2975166 | Amplification | 9377 | 9 | 3 (1.78) | 0 (0) | - | 0.28 | 0 (0) | - | 0 (0) | - | Intergenic | *ARSH* | 4.36 | *ARSF* | 19.69 |
| chrX:38371563-38514390 | Amplification | 142828 | 135 | 3 (1.78) | 0 (0) | - | 0.28 | 0 (0) | - | 1 (7) | *TSPAN7* | Exon | In | 0 | In | 0 |

*^a^* OR comparing PE cases and normotensive controls

*^b^* OR comparing PE cases and schizophrenia study controls

Black box borders denote contiguous merged CNV regions. Genomic positions are designated according to NCBI36/hg18 human genome assembly. Abbreviations: CI, confidence interval; CNV, copy-number variant; OR, odds ratio; PE, preeclampsia.
